# Extracellular vesicles derived from mesenchymal stromal cells: a therapeutic option in respiratory diseases?

**DOI:** 10.1186/s13287-016-0317-0

**Published:** 2016-04-14

**Authors:** Soraia C. Abreu, Daniel J. Weiss, Patricia R. M. Rocco

**Affiliations:** Laboratory of Pulmonary Investigation, Carlos Chagas Filho Institute of Biophysics, Federal University of Rio de Janeiro, Av. Carlos Chagas Filho, 373, Ilha do Fundão, Rio de Janeiro, RJ 21941-902 Brazil; Department of Medicine, Vermont Lung Center, College of Medicine, University of Vermont, 89 Beaumont Ave Given, Burlington, VT 05405 USA

## Abstract

Extracellular vesicles (EVs) are plasma membrane-bound fragments released from several cell types, including mesenchymal stromal cells (MSCs), constitutively or under stimulation. EVs derived from MSCs and other cell types transfer molecules (such as DNA, proteins/peptides, mRNA, microRNA, and lipids) and/or organelles with reparative and anti-inflammatory properties to recipient cells. The paracrine anti-inflammatory effects promoted by MSC-derived EVs have attracted significant interest in the regenerative medicine field, including for potential use in lung injuries. In the present review, we describe the characteristics, biological activities, and mechanisms of action of MSC-derived EVs. We also review the therapeutic potential of EVs as reported in relevant preclinical models of acute and chronic respiratory diseases, such as pneumonia, acute respiratory distress syndrome, asthma, and pulmonary arterial hypertension. Finally, we discuss possible approaches for potentiating the therapeutic effects of MSC-derived EVs so as to enable use of this therapy in clinical practice.

## Background

In recent decades, the therapeutic potential and safety of mesenchymal stromal cells (MSCs) has been studied in the context of regeneration and immune modulation of injured tissues [1]. Many studies have demonstrated that, when systemically administered, MSCs are recruited to sites of inflammation through still-incompletely understood chemotactic mechanisms [2], stimulate endogenous repair of injured tissues [3], and modulate immune responses [4]. The beneficial effects of MSCs on tissue repair and regeneration are based on their paracrine activity, characterized by the capacity to secrete growth factors, cytokines, and chemokines, which orchestrate interactions within the microenvironment and influence tissue regeneration. These factors can inhibit apoptosis, stimulate proliferation, promote vascularization, and modulate the immune response [5]. Remarkably, conditioned medium collected from MSCs can convey many of these protective effects, suggesting that soluble factors rather than cell–cell contact are the major mechanism of MSC actions [6].

**Fig. 1:**
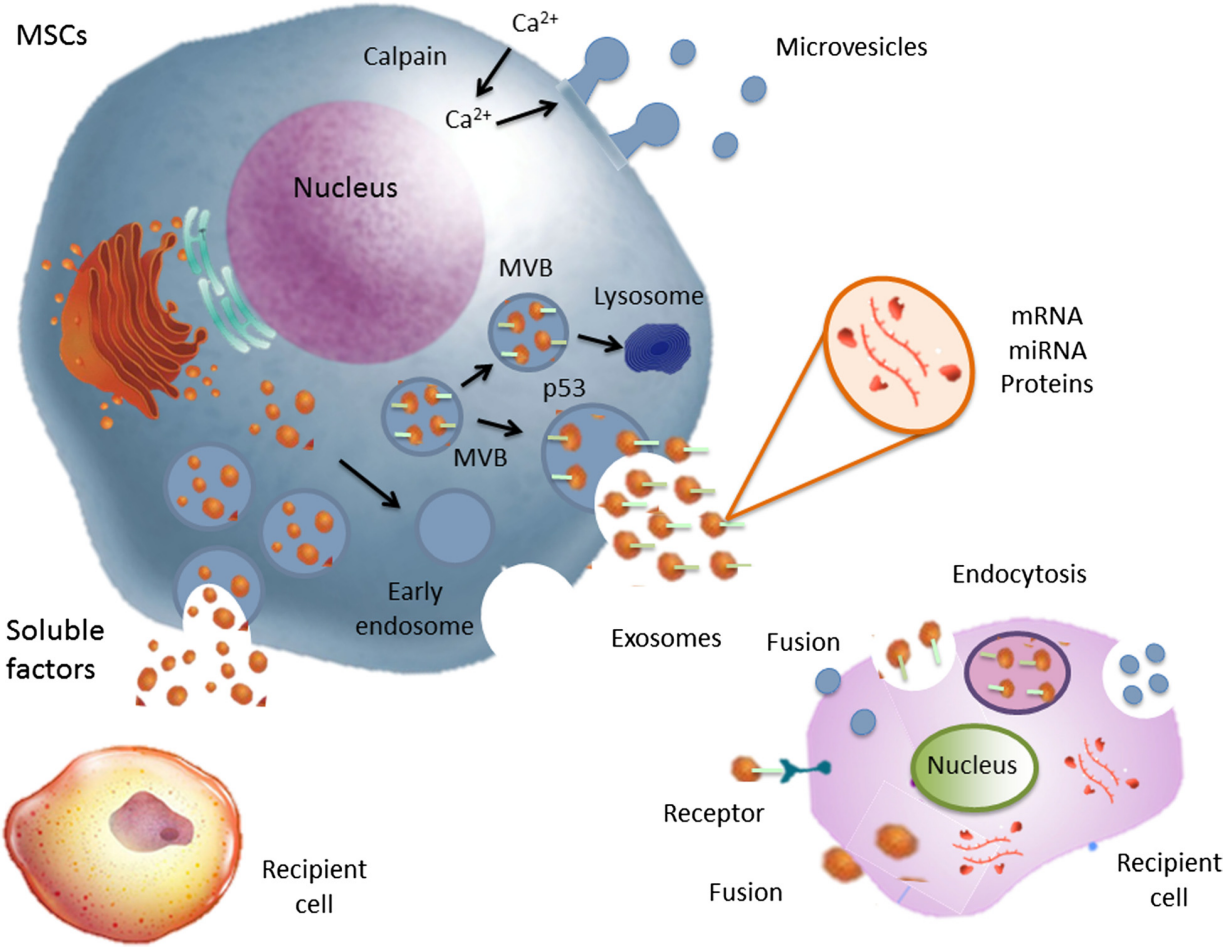
Schematic representation of EVs biogenesis. Vesicles bud directly from the plasma membrane, whereas exosomes originate from ILVs that are generated by inward budding of the limiting membrane of a subgroup of late endosomes called multivesicular bodies (*MVBs*). MVBs can be directed towards the cell periphery and, after fusion with the plasma membrane, release their content into the extracellular space. *miRNA* microRNA, *MSC* mesenchymal stromal cell

Notably, a growing body of literature suggests that many of these paracrine effects are mediated by extracellular vesicles (EVs) contained in the conditioned medium. EVs are small, spherical membrane fragments including exosomes, microvesicle particles, and apoptotic bodies in accordance with the recommendations of the International Society for Extracellular Vesicles (ISEV) [7]. The EVs are released by cells that are involved in cell-to-cell communication and are capable of altering the fate and phenotype of recipient cells [8]. The exosomes arise from intracellular endosomes, while the microvesicles originate directly from the plasma membrane. These particle types are secreted from a wide range of different cell types, including T and B lymphocytes, dendritic cells (DCs), mast cells, platelets, and MSCs derived from different tissues (bone marrow, placenta, as well as adipose and lung tissues), and can also be isolated in vivo from body fluids such as urine, serum, and bronchoalveolar lavage fluid (BALF) [9, 10]. Nevertheless, the classification of EVs differs depending on their origin, size, and contents (Table 1). Additionally, the number and nature of EVs may be affected by gender, age, circadian rhythms, fasting state, medication exposure, and physical activity [11]. However, whether these different classes of EVs represent distinct biological entities is not evident. Several parameters have been used to characterize the different classes of EVs, including size, ionic composition, sedimentation rate, flotation density on a sucrose gradient, lipid composition, protein cargo, and biogenesis pathway; however, most of these parameters are neither definitive nor exclusive to any specific class of EVs (Fig. 1) [7].

**Table 1:** Characterization of extracellular vesicles

Extracellular vesicles	Origin	Size	Content	Markers
Exosomes	Multivesicular bodies	50–150 nm	Proteins, and lipids, DNA, mRNA and miRNA	CD63, CD81, CD9, heat-shock proteins, Alix, Tsg101, integrin, annexins and MHC classes I and II
Microvesicles	Plasma membrane	150–1000 nm	Proteins, and lipids, DNA, mRNA, miRNA and cell organelles.	Integrins, flotillins and tetraspanins
Apoptotic bodies	Membrane of dying cells	**>**1 μm	DNA, noncoding RNAs and cell organelles	Surface markers for macrophages

*Alix* ALG-2-interacting protein X, *MHC* major histocompatibility complex, *miRNA* microRNA, *Tsg101* tumor susceptibility gene 101

Exosomes range in size from 50 to 150 nm, have a homogeneous shape, and are defined as a subtype of EVs derived from specialized intracellular compartments, the multivesicular bodies (MVBs) [12]. Exosomes are constitutively released from cells, but their release is augmented significantly following activation by soluble agonists (cytokines, chemokines, and growth factors), as well as physical, chemical (oxidative stress and hypoxia), and shear stresses [13]. In order to form an exosome, the limiting membrane of the MVBs buds inward, thus forming intraluminal vesicles (ILVs), which then fuse with the plasma membrane to release ILVs as exosomes. This process is mediated by p53-regulated exocytosis, which is dependent on cytoskeletal activation but independent of cell calcium influx [14]. In contrast, microvesicles range from 150 to 1000 nm in size and are more heterogeneous. They are released by budding of small cytoplasmic protrusions, a process dependent on calpain, cytoskeletal reorganization, and intracellular calcium concentration. Calcium ions are responsible for the asymmetric phospholipid distribution of the plasma membrane that yields microvesicle formation [14]. Finally, there is another type of EVs, larger than 1 μm: the apoptotic body, derived from dying cells. DNA, as a residue of the nucleus, is frequently present within these vesicles, as are noncoding RNAs and cell organelles [15].

The different EVs can be isolated from body fluids or in vitro cultured cells by specific standardized protocols, and characterized by differential ultracentrifugation, ultrafiltration, and immunoprecipitation with the use of antibody-loaded magnetic cell beads [16]. These procedures are critical because all types of vesicles, as well as membrane fragments, are normally present in the starting material and can contaminate specific EVs preparations. One major challenge in EVs research is therefore to standardize methods for isolation and analysis. Additionally, it is difficult to distinguish between exosomes and microvesicles because of their overlapping characteristics and the lack of discriminating markers [17]. Nevertheless, among the many subtypes of EVs, exosomes have emerged as physiologically relevant and powerful components of the MSC secretome [18].

The content of EVs consists of proteins, lipids, and nucleic acids; microvesicles and apoptotic bodies also have organellar contents. Since the effects of EVs usually depend on their cell of origin and may be influenced by physiological stress or pathological conditions, they could be used as biomarkers to diagnose, prognosticate, or predict diseases and their natural history [14]. Many reports have shown that the functions of EVs reflect, at least in part, those of their originating cells; differences between them occur because the EVs composition may be modified, suggesting that preferential packaging or exclusion of material occurs [19]. Information on the protein, lipid, and RNA expressions of EVs is collected in VESICLEPEDIA (http://www.microvesicles.org) [20], while the exosomes of different cell types and organisms are described in the ExoCarta database [21]. EVs play an important role in intercellular communication and are capable of modifying the activity of target cells through direct surface receptor interactions, receptor transfer between cells, protein delivery to target cells, or horizontal transfer of genetic information [22]. They are involved in cellular processes such as angiogenesis modulation, cell proliferation, and immune regulation [23]. EVs are therefore particularly attractive for their therapeutic potential, especially MSC-derived EVs, which appear to be an important tool to harness the clinical benefits of MSC therapy while using cell-free strategies based on the MSC secretome. These strategies may reduce the risks associated with engraftment of MSCs, such as possible immune reactions against MSCs and development of ectopic tissue. Since EVs carry a wide array of signals, several studies have been performed evaluating their implication in animal models of organ injury, including lung diseases. Nonetheless, comprehensive insight regarding the full scope of molecules packaged in MSC-derived EVs and their role in tissue regeneration has yet to be gained, and additional studies are needed to provide greater detail [9, 23].

## Characteristics of MSC-derived EVs

MSC-derived EVs express surface molecules, such as CD29, CD73, CD44, and CD105, which are characteristic of their cells of origin. Among the MSC-derived EVs, the exosomes are those best characterized. Exosomes are known to conserve a set of proteins, including tetraspanins, involved in cell targeting (CD63, CD81, and CD9); heat-shock proteins Hsp60, Hsp70, and Hsp90 [24]; ALG-2-interacting protein X (Alix) and tumor susceptibility gene 101 (Tsg101), which are involved in their biogenesis from MVBs; integrins and annexins, which are important for transport and fusion [20]; and major histocompatibility complex classes I and II [25]. Microvesicles lack proteins of the endocytic pathway, but are rich in cholesterol and lipid raft-associated proteins, such as integrins and flotillins. Although tetraspanins are commonly used as unique markers for exosomes, they can be detected in microvesicles in some cases [26]. Several studies have been conducted evaluating the potential role of MSC-derived EVs in physiological and pathological conditions and their possible applications in the therapy of different diseases [12, 15]; however, few studies have evaluated the RNA and protein content of these vesicles.

MSC-derived EVs are enriched by distinct classes of RNAs that could be transferred to target cells and translated into proteins, resulting in an alteration of target cell behavior [27]. In particular, MSC-derived EVs contain transcripts involved in control of transcription (transcription factor CP2, clock homolog), cell proliferation (retinoblastoma-like 1, small ubiquitin-related modifier 1), and immune regulation (interleukin 1 receptor antagonist) [27]. Additionally, MSC-derived EVs contain noncoding RNA, microRNAs (miRNAs) that mediate posttranscriptional control of gene expression and, as such, modulate survival and metabolic activities of recipient cells [28]. These miRNAs can be present both in EVs and/or in their cells of origin [9]. The miRNAs detected in MSC-derived EVs are usually related to development, cell survival, and differentiation, while some MSC-derived EVs-enriched miRNAs are more closely associated with regulation of the immune system [9]. Comprehensive information on the complete RNA content of MSC-derived EVs is not currently available, however, and whether adult MSCs from different sources share similar RNA repertoires remains unknown. A recent study compared the RNA profile of exosomes released by adult MSCs from two different sources: adipose-derived MSCs (ASCs) and bone marrow-derived MSCs (BM-MSCs). Despite substantial similarity between the most represented RNAs in the ASC and BM-MSC exosomes, their relative proportions are different [29].

Proteome analysis may be equally important. Characterization of the content of BM-MSC-derived EVs identified several proteins, among which are mediators controlling self-renewal and differentiation. Interestingly, this analysis revealed a number of surface markers, such as platelet-derived growth factor receptor, epidermal growth factor receptor, and plasminogen activator, urokinase receptor; signaling molecules of the RAS-mitogen-activated protein kinase, Rho GTPase, and Cell division control protein 42 pathways; cell adhesion molecules; and additional MSC antigens [30], supporting a possible role for such vesicles in tissue repair. Treatment of cell-derived EVs with specific growth factors can change the phenotype and protein content of these vesicles; for example, ASCs treated with platelet-derived growth factor have been shown to produce EVs with enhanced angiogenic activity [31]. This wide distribution of biological activities gives MSC-derived EVs the potential to elicit diverse cellular responses and interact with many cell types.

## Mechanisms of action and biological activities of EVs

EVs may interact with recipient cells by different mechanisms: interactions at the cell surface, internalization into endocytic compartments, and fusion with plasma membranes (Fig. 1) [32]. The efficiency of EVs uptake has been observed to correlate directly with intracellular and microenvironmental acidity [33]. Following ligand interaction, EVs may deliver their contents to the recipient cell that reprogrammed them. Recently, EVs from stem cells were demonstrated to shuttle a cysteine-selective transport channel (cystinosin) that restores function in mutant target cells [34]. EVs may also mediate the horizontal transfer of genetic information, such as subsets of mRNA and miRNA, from the cell of origin, thereby inducing alterations in the phenotype and behavior of recipient cells by different pathways [35]. In this line, EVs produced by murine embryonic stem cells may reprogram hematopoietic progenitors by delivering not only proteins but also mRNA for several pluripotent transcription factors [36], whereas pretreatment of these EVs with RNase inhibited the observed biological effects, thus suggesting the contribution of EVs-derived mRNA [36]. Stem cells may therefore modulate their biological effects by delivering genetic information and altering the gene expression of target cells. Interestingly, the exchange of genetic information may be bidirectional: from injured cells to bone marrow-derived or resident stem cells; or from stem cells to injured cells. In this context, Dooner et al. [37] reported that bone marrow stem cells cocultured with injured lung cells expressed genes for lung-specific proteins, such as surfactant B and C, and Clara cell-specific proteins, which may be attributed to the transfer of lung-specific mRNAs to bone marrow cells via EVs released from the injured lung cells.

Additionally, EVs derived from injured and immune cells may induce stem cell recruitment and differentiation of resident stem cells present in several organs during adulthood, thus contributing to physiologic tissue repair [13]. Nevertheless, depending on their cells of origin, EVs can exert immunostimulatory or immunosuppressive effects [38]. Alveolar macrophages infected with mycobacteria release EVs containing pathogen-derived proinflammatory molecules and secrete Hsp70, which activates the nuclear factor-κB pathway by stimulating toll-like receptors (TLRs) [15], leading to the secretion of proinflammatory cytokines [14, 24]. On the other hand, EVs secreted by DCs are able to induce humoral responses against antigens processed by DCs before EVs purification, yielding strong protection against infection [39]. EVs may also modulate the function of target cells. For instance, EVs derived from lipopolysaccharide-activated monocytes induce apoptosis in target cells through transfer of caspase-1 [40]. Furthermore, proteomic analysis of damaged tissues usually reveals they are depleted of many rate-limiting ATP-generating enzymes, and are thus unable to utilize the restored oxygen supply to produce ATP. This depletion could be supplemented by the proteome of MSC-derived exosomes, which has a cargo rich in enzymatically active glycolytic enzymes and other ATP-generating enzymes, such as adenylate kinase and nucleoside-diphosphate kinase [41].

**Fig. 2:**
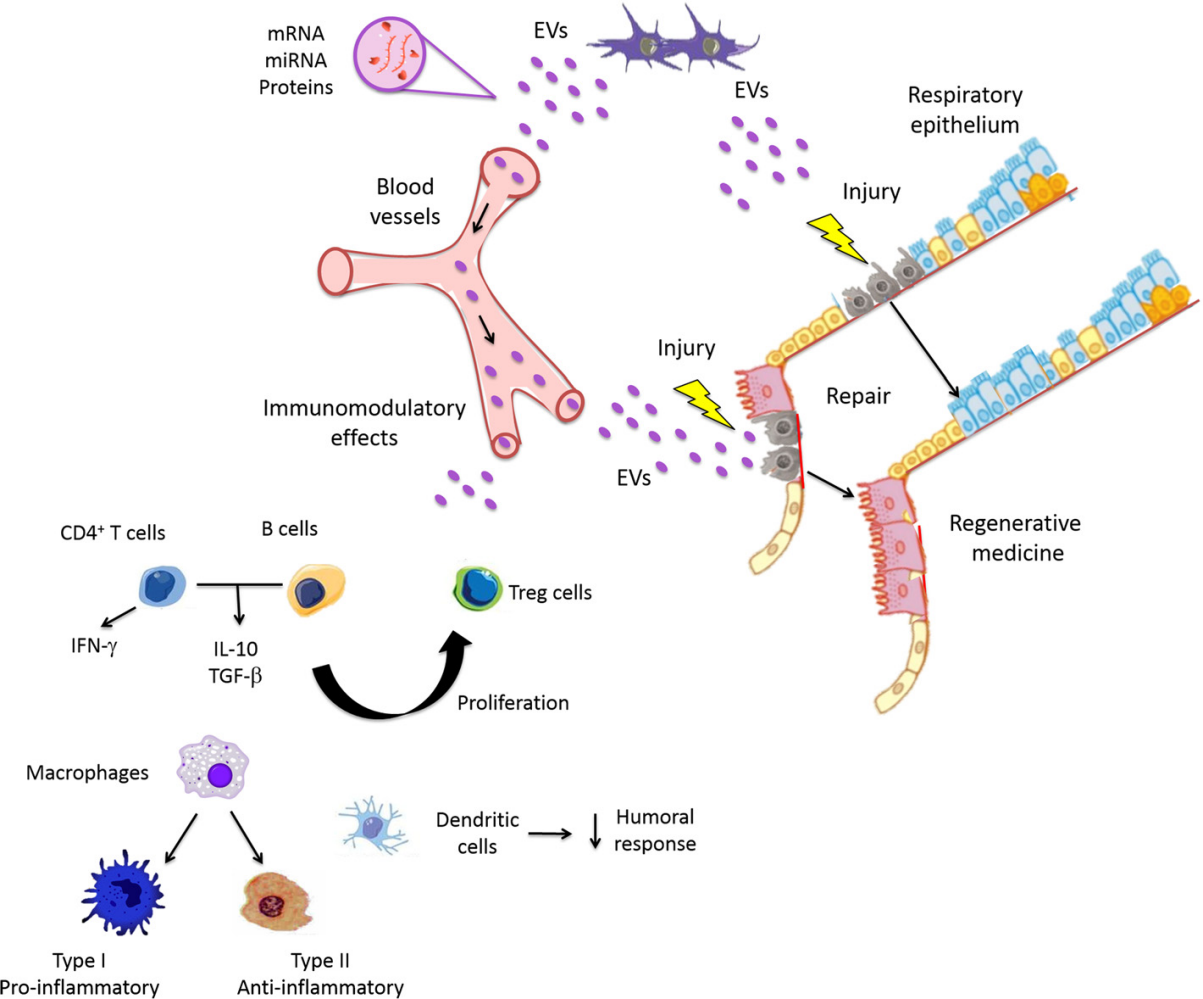
Scheme illustrating extracellular vesicle (*EV*) function related to tissue repair. The exchange of proteins and genetic information (mRNA and miRNA) from MSCs or resident stem cells contributes to tissue repair. *IFN* interferon, *IL* interleukin, *miRNA* microRNA, *TGFβ* transforming growth factor beta, *Treg* regulatory T cell

However, MSC-derived EVs have received more emphasis in the literature and have been most widely studied. In this line, EVs released from human MSCs have been shown to contain ribonucleoproteins involved in the intracellular trafficking of RNA and selected patterns of miRNA, suggesting dynamic regulation and compartmentalization of RNA involved in the development, regulation, regeneration, and cell differentiation, which contribute to recovery processes after injury of adult tissues (Fig. 2) [42]. Indeed, MSC-derived EVs exert an important inhibition in the differentiation and activation of T cells and their interferon-gamma (IFN-γ) release in vitro, as well as stimulating the secretion of anti-inflammatory cytokines (interleukin (IL)-10 and transforming growth factor beta (TGF-β)) and generation of regulatory T cells [43], suggesting that MSC-derived exosomes are relevant immunomodulatory therapeutic agents (Fig. 2). Additionally, treatment with MSC-derived EVs activates an M2 macrophage-like phenotype in the lung parenchyma, which is known to promote tissue repair and limit injury [44].

The immunomodulatory effects of BM-MSCs and derived EVs have been analyzed in vitro. BM-MSCs and their EVs exhibit similar inhibitory activity against B-cell proliferation, but EVs display less inhibitory activity on differentiation and antibody release of B cells compared with BM-MSCs. Moreover, BM-MSCs are more efficient than EVs at inhibiting T-cell proliferation. In one study, incubation of both T cells and B cells with EVs led to a decrease in granulocyte–macrophage colony-stimulating factor and IFN-γ and an increase in IL-10 and TGF-β compared with BM-MSCs [45].

## Therapeutic potential of MSC-derived EVs in lung diseases

MSC-derived EVs have shown to be a promising therapy enabling tissue repair and wound healing. The effects of MSC-derived EVs can be potentiated under some conditions, such as exposure to hypoxia and coculture with animal or human serum obtained in pathologic conditions. These methods may induce the release and potentiate the effects of these EVs due to stimulation and the presence of cytokines and chemotactic and growth factors, which not only increase EVs release but may also modify their content, leading to enhancement of beneficial effects.

EVs are also important vehicles for drug delivery because of their lipid bilayer and aqueous core, since they can carry both lipophilic and hydrophilic drugs [46]. Furthermore, EVs present several advantages for this purpose, such as: presence of protein and genetic materials, which enables active loading of biological material; high tolerability in the body due to the presence of inhibitors of complement and phagocytosis [30]; protection against degradative enzymes or chemicals; and ability to cross the plasma membrane to deliver their cargo to target cells [9, 47] and home to target tissues [9, 46]. Electroporation [48] and viral packaging strategies [49] have been used to load therapeutically active cargo molecules (e.g., small-molecule drugs or small interfering RNA (siRNA)) into EVs [48, 49].

While a predominant mechanism of MSCs in tissue repair through paracrine activity has already been suggested, some studies are being carried out to better understand the mechanisms associated with the beneficial effects of MSC-derived EVs in lung diseases, such as asthma, pulmonary arterial hypertension (PAH), acute respiratory distress syndrome (ARDS), and pneumonia (Table 2), and how they can be potentiated for translation to clinical practice.

**Table 2:** Effects of extracellular vesicles in lung diseases

Study	Model	Origin	Effects
Admyre et al., 2008 [55]	Allergic inflammation	Mast cell-derived EVs	DC maturation, allergen transportation, allergen-specific Th2 cell activation
Bakouboula et al., 2008 [63]	PAH	EVs released from stimulated or endothelial cells undergoing apoptosis	Increase in EVs release is directly related to PAH severity
Prado et al., 2008 [61]	Allergic inflammation	BALF-derived EVs from mice sensitized and challenged with ovalbumin	Inhibition of IgE response, Th2 cytokine production, and airway inflammation
Ionescu et al., 2012 [70]	Endotoxin-induced ARDS	CM from MSC	Increase in secretion of exosomes by MSCs and M2 macrophages, in part via IGF-1
Lee et al., 2012 [65]	Hypoxia-induced PAH	MSC-derived EVs	Reduced right ventricular systolic pressure and right ventricular hypertrophy
Torregrosa et al., 2012 [60]	Coculture of BECs with BALF EVs from asthmatic patients	EVs from BALF of asthmatic patients	Increased leukotriene and IL-8 release
Aliotta et al., 2013 [64]	Monocrotaline-induced PAH	Lung-derived and plasma-derived EVs from monocrotaline-induced PAH	Increased right ventricular mass and pulmonary vascular wall thickness
Zhu et al., 2014 [72]	*Escherichia coli* endotoxin-induced ARDS	EVs derived from hMSCs	Reduction in extravascular lung water, total protein levels in BALF, edema, neutrophil infiltration, associated with increased KGF expression
Cruz et al., 2015 [62]	*Aspergillus* hyphal extract-induced allergic inflammation	CM and EVs derived from hMSCs and mMSCs	More significant reduction of airway hyperresponsiveness, lung inflammation and CD4 T-cell Th2 and Th17 phenotype in both CM and EVs from hMSCs compared with mMSCs; inhibition of soluble mediators and EVs release reduced the beneficial effects of all treatments
Monsel et al., 2015 [78]	*Escherichia coli* pneumonia	MSC and MSC-derived EVs	Improved survival and reduced lung inflammation, protein permeability, and bacterial growth

*ARDS* acute respiratory distress syndrome, *BALF* bronchoalveolar lavage fluid, *BEC* bronchial epithelial cell, *CM* conditioned medium, *DC* dendritic cell, *EV* extracellular vesicle, *hMSC* human mesenchymal stem cell, *IGF-1* insulin-like growth factor-1, *IL* interleukin, *KGF* keratinocyte growth factor, *MSC* mesenchymal stem cell, *mMSC* mouse mesenchymal stem cell, *PAH* pulmonary arterial hypertension, *Th* T-helper

### Asthma

Asthma is a chronic inflammatory disease characterized by airway constriction and inflammation, which may lead to structural changes in the airways, often in response to allergens, infections, and air pollutants [50]. Even though several therapeutic strategies are currently available to reduce airway inflammation, no treatment has so far been able to hasten repair of the damaged lung [51]. In this line, some studies reported that MSCs reduced lung inflammation and remodeling in experimental allergic asthma [52–54].

EVs are released from several cells that are involved in allergies, including mast cells, DCs, T cells, and bronchial epithelial cells (BECs) in the lungs. For example, mast cell-derived EVs induce DC maturation, and DC-derived EVs can transport allergens and activate allergen-specific T-helper (Th) type 2 cells [55]. Among several potential mechanisms, BECs exposed to compressive stress – thus simulating the bronchoconstriction seen in asthma – produce EVs bearing tissue factor which may participate in promotion of subepithelial fibrosis and angiogenesis [56]. In short, available data indicate the potential contributions of BEC-derived EVs to the pathogenesis of asthma. Additionally, these findings may lead to the development of future treatments for asthma patients that target the inhibition of EVs secretion by these cells.

Several phenotypic and functional alterations have been observed in BALF EVs from asthmatics compared with healthy patients. These include higher expression of CD36, which has been implicated in bacterial recognition and may play a role in asthma exacerbations in response to bacterial infections [57], and that the EVs contain miRNAs, critical regulators of specific pathogenic events [58] which may act as biomarkers of lung diseases, such as the let-7 (let-7a–let-7e) and miRNA-200 (miR-200b and miR-141) families [59]. Further, incubation of BECs with BALF EVs from asthmatic patients resulted in increased leukotriene and IL-8 release [60].

Additionally, administration of BALF-derived EVs from mice sensitized and challenged with ovalbumin has been shown to inhibit IgE response, Th2 cytokine production, and airway inflammation in experimental asthma [61]. Similar behavior was observed with asthmatic serum-derived EVs, protecting against allergic airway inflammation and reducing BALF eosinophil counts, IgE levels, and Th2 response. EVs from different sources may therefore play a role in the development of asthma and allergy, either as a failure to induce effective tolerance or as enhancers of an already established response. In short, EVs may be therapeutic targets in anti-allergy treatment.

Recently, the therapeutic effects of EVs derived from human MSCs (hMSCs) and mouse MSCs (mMSCs) were investigated in experimental asthma. The authors observed that systemic administration of EVs from either hMSCs or mMSCs were each effective – in some cases, more effective than the administration of hMSCs or mMSCs themselves – in mitigating allergic airway hyperresponsiveness and lung inflammation, and altered the phenotype of antigen-specific CD4 T cells in a model of severe, acute, mixed Th2/Th17-mediated eosinophilic and neutrophilic airway allergic inflammation in immunocompetent mice. Additionally, blocking EVs release led to an absence of protective effects associated with both hMSCs and mMSCs [62].

### Pulmonary arterial hypertension

PAH is a disease characterized by hyperplasia and hypertrophy of smooth muscle cells in small pulmonary arteries, associated with an increase in endothelial cell proliferation that leads to remodeling of pulmonary vessels and, consequently, an increase in mean pulmonary arterial pressure and right ventricular overload. Data obtained from patients with PAH show that the severity of PAH is related to an increase in circulating EVs released from stimulated or endothelial cells undergoing apoptosis, probably due to release of soluble vascular cellular adhesion molecule VCAM-1, and that proinflammatory markers, such as monocyte chemoattractant protein MCP-1 and highly specific C-reactive protein, were elevated in PAH patients. In addition, a further increase in endothelium-derived CD105 microparticles was observed in pulmonary arterial blood compared with venous blood in patients with PAH [63]. Inflammation plays an important role in the development of human PAH and there are several animal models of this condition, such as monocrotaline-induced and hypoxia-induced PAH in rodents.

Despite significant progress in elucidating PAH pathophysiology and treatment, few PAH therapies are available and all have limited effectiveness. Many studies have therefore investigated the effects of MSC therapy in PAH, and demonstrated benefit. In a recent investigation, lung-derived and plasma-derived EVs generated from monocrotaline-induced PAH led to increased right ventricular mass and pulmonary vascular wall thickness, resulting in PAH-like changes in healthy mice This effect may be promoted directly by EVs on the pulmonary vasculature or by differentiation of bone marrow cells to endothelial progenitor cells that induce pulmonary vascular remodeling [64]. This suggests that EVs presented altered expressions of miRNAs involved in pulmonary vascular remodeling. Conversely, in hypoxia-induced PAH, MSC-derived EVs protected against elevation of right ventricular systolic pressure and development of right ventricular hypertrophy, whereas EVs-depleted medium and fibroblast-derived EVs had no effect. These beneficial effects of MSC-derived EVs can be related to suppression of hypoxic pulmonary macrophage influx and hypoxic activation of signal transducer and activator of transcription STAT3, combined with induction of proinflammatory and proproliferative mediators – including MCP-1 and hypoxia-inducible mitogenic factor HIMF – and increased pulmonary levels of the key miRNAs miR-17 and miR-204, the expressions of which are reduced in human pulmonary hypertension [65]. However, the animal models in which these effects were tested are not considered good representations of preclinical models of PAH. The beneficial effects observed with EVs treatment of PAH therefore require more in-depth investigation before they can be considered practice changing.

### Acute respiratory distress syndrome

ARDS is a severe clinical condition characterized by alveolar-capillary damage, accumulation of protein-rich debris in the alveolar airspace, and progressive respiratory failure [66]. Although major improvements in treatment and supportive care of ARDS have been achieved, its mortality rate remains around 40 % [67].

Recently, some studies reported that MSCs can be a promising therapeutic approach for ARDS through paracrine effects [68–70]. Additionally, MSC-derived EVs have been shown to produce beneficial effects in experimental endotoxin-induced ARDS, reducing lung inflammation [71]. hMSC-derived EVs were therapeutically effective following *Escherichia coli* endotoxin-induced ARDS, thus reducing extravascular lung water, total protein levels in BALF, edema, and neutrophil infiltration. These beneficial effects were associated with an increase in keratinocyte growth factor (KGF) expression, as they were partially eliminated after delivery of EVs derived from KGF siRNA-pretreated MSCs [72]. Moreover, ischemic preconditioning can potentiate the protective effect of MSCs in endotoxin-induced ARDS through the secretion of exosomes since it confers strong protection against cell death and promotes their differentiation potential by activating multiple signaling pathways which open new avenues for therapeutic approaches [73].

### Pneumonia

Bacterial pneumonia is among the main causes of respiratory failure in critically ill patients. Despite improvements in supportive care and appropriate antibiotic use, morbidity and mortality remain high [74]. Several studies have reported efficacy of MSCs in preclinical models of pneumonia due to their ability to secrete paracrine factors such as growth factors, anti-inflammatory cytokines, and antimicrobial peptides [75]. Outer-membrane vesicle release is a conserved phenomenon among pathogenic and nonpathogenic Gram-negative bacteria [76]. Nevertheless, little is known regarding Gram-positive EVs, especially their biogenesis and role in host–pathogen interactions. EVs from *Streptococcus pneumonia*, one of the leading causes of bacterial pneumonia worldwide, have only recently been characterized [77] and found to exhibit high immunogenicity due to the presence of the toxin pneumolysin.

Recently, in an in vivo model of *E. coli* pneumonia in mice, hMSC-derived EVs were as effective as their parent stem cells in improving survival and mitigating lung inflammation, protein permeability, and bacterial growth. The antimicrobial effect of hMSC-derived EVs was exerted in part through enhancement of monocyte phagocytosis of bacteria, which could be further increased by prestimulation of hMSCs with a TLR-3 agonist before EVs release. Uptake of hMSC-derived EVs through the CD44 receptor into injured human monocytes and alveolar epithelial cells was critical for their therapeutic effects. Another factor that should be stressed is that hMSC-derived EVs decreased tumor necrosis factor alpha secretion by lipopolysaccharide-primed human monocytes and restored intracellular ATP levels in injured human alveolar epithelial type II cells, suggesting immunomodulatory and metabolomic effects of EVs. Additionally, administration of a KGF neutralizing antibody abrogated the survival advantage mediated by hMSC-derived EVs, suggesting a possible mechanism for their therapeutic effect [78].

## Conclusions

Several studies have reported that MSCs may repair damaged tissue by modifying target cell function through paracrine mechanisms without directly replacing injured cells. The role of EVs in this mechanism would be to exchange genetic material, which could explain the observed phenotypic and functional changes of MSCs [79]. This genetic material transfer may lead to the production of soluble factors, thus regulating cell proliferation, apoptosis, and/or inflammation and immune response.

EVs present many advantages over stem cells, such as homing ability to target tissue, preventing undesired accumulation in other organs, and absence of any innate toxicity or association with long-term maldifferentiated engrafted cells, tumor generation, or immune rejection after stem cell injection. However, the mechanisms associated with the beneficial effects induced by MSC-derived EVs require further investigation. In this line, the following points in particular warrant better evaluation: which signaling regulates the transfer of biologically active molecules within EVs, which surface receptors may yield selective specificity, and which stimuli are responsible for triggering EVs release. Understanding these EVs mechanisms may allow their use as diagnostic markers, for the delivery of drugs and genes, and as new therapeutic strategies. Although some studies have reported beneficial effects of MSC-derived EVs in asthma, ARDS, PAH, and pneumonia, many issues must be addressed before their use in clinical settings, including: the need for large-scale EVs production from MSCs; the need for criteria defining the potency of EVs, due to different preparations and MSC sources; the long-term effects of EVs; and the biodistribution of EVs in each respiratory disease.

## Abbreviations

ARDS: Acute respiratory distress syndrome
ASC: Adipose-derived mesenchymal stromal cell
BALF: Bronchoalveolar lavage fluid
BEC: Bronchial epithelial cell
BM-MSC: Bone marrow-derived mesenchymal stromal cell
DC: Dendritic cell
EV: Extracellular vesicle
hMSC: Human mesenchymal stromal cell
Hsp: Heat-shock proteins
IFNγ: Interferon gamma
IL: Interleukin
ILV: Intraluminal vesicle
ISEV: International Society for Extracellular Vesicles
KGF: Keratinocyte growth factor
miRNA: MicroRNA
mMSC: Mouse mesenchymal stromal cell
MSC: Mesenchymal stromal cell
MVB: Multivesicular body
PAH: Pulmonary arterial hypertension
siRNA: Small interfering RNA
TGF-β: Transforming growth factor beta
Th: T-helper
TLR: Toll-like receptor
